# Implementation of national guidelines, incorporated within structured diabetes and hypertension records at primary level care in Cape Town, South Africa: a randomised controlled trial

**DOI:** 10.3402/gha.v6i0.20796

**Published:** 2013-09-25

**Authors:** Krisela Steyn, Carl Lombard, Nomonde Gwebushe, Jean M. Fourie, Katherine Everett-Murphy, Merrick Zwarenstein, Naomi S. Levitt

**Affiliations:** 1 Department of Medicine, Chronic Disease Initiative for Africa, University of Cape Town, Cape Town, South Africa; 2 Chronic Diseases of Lifestyle Unit, Medical Research Council, Tygerberg, South Africa; 3 Biostatistics Unit, Medical Research Council, Tygerberg, South Africa; 4 Department of Family Medicine, Centre for Studies in Family Medicine, Western University, London, Ontario, Canada

**Keywords:** implementation, clinical guidelines, diabetes, hypertension, primary care

## Abstract

**Background and objectives:**

Many clinical management guidelines for chronic diseases have been published, but they have not been put into practice by busy clinicians at primary care levels. This study evaluates the implementation of national guidelines incorporated within a structured diabetes and hypertension clinical record (SR) in Cape Town in a randomised controlled trial (RCT).

**Methods:**

Eighteen public sector community health centres (CHC) were randomly selected and allocated as intervention or control CHC. At each clinic, 25 patients with diabetes and 35 patients with hypertension were enrolled at baseline. Questionnaires were completed, blood samples were collected, blood pressure (BP) and anthropometric measures were taken and patient records were audited. SR with clinical guideline prompts were introduced at the intervention clinics after training doctors in their use and suggestions to incorporate them in regular patient records. Contact was maintained during the year of intervention with the clinic staff. A follow-up survey was conducted 1 year later to assess BP and HbA1c, and the patient records were examined to ascertain the extent of use of the SR in the intervention clinics. In-depth interviews were conducted with doctors and nurses to record their response to the intervention.

**Results:**

The intervention evaluated in this RCT had no impact on either diabetes or hypertension control. In the intervention clinics, less than 60% of the patient folders contained the SR and when present was seldom used. Although the staff were well disposed to the research team, their workload prohibited them from undertaking a true evaluation of the SR, and overall they did not perceive the SR as supporting their current process of patient care.

**Conclusions:**

No benefit to diabetes of hypertension care by introducing and availability of the staff in the use of the SR was shown in this RCT. The process measures suggest that the SR was not widely used by the healthcare provided in the primary care clinics.

Throughout the world, diabetes and hypertension are major and in many cases, growing health problems. South Africa is no exception to this with its diabetes and hypertension prevalence being the highest in sub-Saharan Africa. (1) Although diabetes and hypertension are associated with considerable morbidity and premature mortality, there is unequivocal evidence that specific aspects of good quality care can improve outcomes. For example: in people with diabetes, good glycaemic control can prevent or retard the development of microvascular complications (2, 3); improved blood pressure (BP) control can reduce both micro and macrovascular complications (4); and screening for retinopathy and peripheral neuropathy with resultant timeous intervention can prevent blindness or lower extremity amputations (5, 6). Consequently, optimal diabetes and hypertension care have become the goal of healthcare providers everywhere.

In general, the management of type 2 diabetes and hypertension takes place at primary care level. In South Africa, most people with these conditions receive their routine care at state-funded primary care clinics. An external audit conducted in such clinics in Cape Town demonstrated considerable deficiencies in the quality of care provided (2). These included substantial proportions of people with poor glycaemic control (50.6%) and inadequate BP control (71.5%) as well as infrequent documented eye and foot examinations despite their common presence found by the researchers. For example, although retinopathy of all grades was present in 55.4% of patients and an abnormality on foot examination present in 36.6% of patients, there were no documented examinations for the former and very low numbers of the latter over the preceding year. It has also been shown that patients with uncontrolled hypertension attending both public and private primary care settings in the townships around Cape Town have higher levels of end organ damage than those with better-controlled hypertension (3).

Clinical practice guidelines have been developed by numerous national and international organisations to assist practitioners in their decision making and appropriate care for patients with many conditions, including diabetes and hypertension. Undeniably, guidelines can change clinical practice and affect outcome, but their success depends on a number of factors such as the methods of their development, dissemination, and implementation as well as the healthcare context (4). In South Africa, the National Diabetes Advisory Board developed national consensus guidelines for the management of type 2 diabetes at primary care level with the input of multiple stakeholders (5). Similarly, the Hypertension Society of Southern Africa developed guidelines for hypertension (6). These guidelines were accepted and disseminated by the National Department of Health. Yet a qualitative and observational study among primary healthcare doctors and professional nurses in Cape Town revealed ambivalence to and infrequent consultation of the national guidelines for the management of diabetes and hypertension, a situation described in other countries (7).

Although, as described by Oxman et al. (8), there is ‘no magic bullet’ to improve professional practice and patient outcome, there are many available interventions that can lead to improvements in care. Oxman et al. list the following possible interventions for improved outcome: the restructuring of medical records; outreach visits; use of local opinion leaders; quality improvement audits and feedback. We undertook an open, cluster RCT (randomised controlled trial) of the effects of introducing a structured clinical record (with the national guidelines imbedded in it) and training of healthcare providers in its use, on the control of diabetes and hypertension. We also assessed the effects of the intervention on the quality of care.

## Methods

### Setting

The study took place in public sector primary healthcare clinics known as Community Health Centres (CHCs) in Cape Town in 1999 and 2000. These CHCs are located in working-class residential areas and provide a network of accessible and free primary care for acute and chronic illnesses. General practitioners and nurses who consult patients with chronic disorders, in the absence of acute illnesses, at 3-, 4-, or 6-monthly intervals, staff the CHCs. Only CHCs with a minimum of 25 patients with diabetes and 35 with hypertension on their registers were eligible for inclusion for randomisation.

### Patients

Patients who attended the clinics for routine care were selected on consecutive days until the required number was recruited. Inclusion criteria were: being 15 years or older, a documented attendee at the particular CHC with at least four visits during the previous year for hypertension or diabetes, and having received treatment for these conditions at each visit. Participants unable to provide answers to a questionnaire were excluded. The same patients were followed up a year later.

### Multifaceted intervention

A structured record (SR), which incorporated the National Guidelines for the management of patients with diabetes or hypertension or both conditions, was designed (7). The SR was a three-sided, folded, A3-sized coloured sheet of paper, which was to be placed in the folder of each patient. Multiple components were included: the front page had a space for the patient's general details, tick blocks for medical history, referrals in the past year, and a list of educational topics to be covered during their clinic visits. The second page provided an algorithm for the diagnosis and management of type 2 diabetes and hypertension, body mass index (BMI) targets, and the names and doses of the available oral hypoglycaemic or BP-lowering agents. The third page included a flow sheet for the record of blood glucose, BP, relevant symptoms, and clinical examination including foot, eye, urine, biochemistry, and specific prompts were provided for certain clinical findings.

An educational package consisted of an outreach visit by a recognised local diabetes and hypertension expert to: (a) review their respective national guidelines, (b) train clinicians in the use of the guideline-based SR, and (c) make suggestions as to the positioning of the SR in the folder. Two further visits took place; the first 2 weeks after an introduction of the guidelines in order to identify problem areas encountered by the clinicians in the use of the SR and the second one, 2 months later. Subsequently, a fieldworker maintained contact with the nursing staff in each clinic to ensure that there were adequate supplies of SR.

Additional SR forms remained available in the clinics until the follow-up survey was completed.

The control arm received usual care, which included the guidelines passively disseminated by the National Department of Health.

### Outcome measures

The primary outcome measures were the mean level of glycated haemoglobin in all patients with diabetes. For patients in the hypertension group, the main outcome measures were the mean systolic and mean diastolic BP in all those with hypertension as measured at the end of the intervention period of 1 year. Secondary outcome measures were the proportion of patients in the hypertension group with controlled BP (BP≤140/90 mmHg and in patients with diabetes BP≤130/85 mmHg) in the hypertension group and uncontrolled glycaemia (percentage with HbA1c ≥7%) in the diabetes group. Quality of care measures were the proportions of patients with recorded examinations for complications (retinopathy, nephropathy, foot problems). The number of patient folders that contained the SR and the degree to which SR was completed was also examined.

### Sample size

The sample size was calculated for each of the primary outcomes separately. For the diabetes outcome, the sample size was calculated based on an expected decline in mean glycated haemoglobin of 1.5% more in the experimental than the control arm with a significance level of 5% and a power of 80%. An inter-cluster correlation of 0.1 was assumed, based on our previous local studies of these facilities (2) and a standard deviation of 3%. We estimated that 18 facilities each with 25 patients with diabetes would be required. For the hypertension outcome, the mean reduction in systolic BP as a result of the intervention was 5 mmHg (standard deviation 10 mmHg) with the same intra-class correlation coefficient, significance level, and power. We estimated that 18 facilities each with 35 patients with hypertension were needed.

### Randomisation

**Fig. 1:**
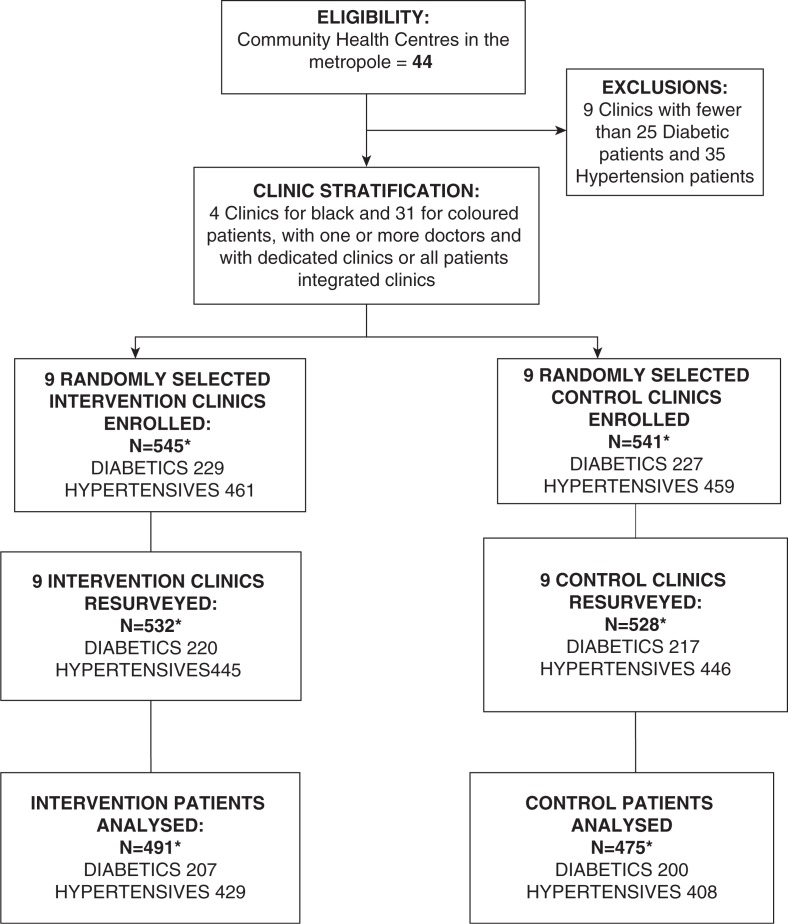
Patient participation in the trial. *284 patients had both diabetes and hypertension.

Figure 1 provides an outline of the trial with the numbers of clinics and patients involved in the study. The 35 eligible clinics were stratified according to: (a) cultural group (*n*=2); (b) single or multiple medical practitioners; and (c) care provided in a dedicated diabetes of hypertension club clinic or with patients seen as part of all patients attending the clinic. We randomly selected two of four eligible clinics serving African patients, and 16 of 31 serving coloured areas from within the other strata to reflect the number of such clinics of each group in the Cape Town Metropole. Study clinics were randomly allocated, by stratum, to intervention or control using a computer-generated list of random numbers. At each clinic, 25 patients with diabetes and 35 patients with hypertension were enrolled on consecutive clinic days.

### Data collection

The baseline data were collected for each clinic over a 1- to 2-month period, depending on the flow of diabetic patients, and the intervention began immediately thereafter. Recruitment of clinics, collection of baseline data, and initiation of the intervention was staggered over a 9-month period, as was follow-up data collection.

Trained fieldworkers conducted interviews with patients and completed pre-coded questionnaires, previously piloted and translated into Afrikaans and isiXhosa. The data recorded included socio-demographic details, clinical history, and patient satisfaction with the clinic services. All participating patients’ medical records of the preceding year were reviewed. A checklist of items, which clinic staff had recorded, reflected aspects of appropriate care for the patients. At follow-up, fieldworkers took note of the proportion of patients whose folders contained SR forms and recorded the extent to which the SRs were completed.

Height, weight, and BP were measured and BMI was calculated. Defined BP control recommended a target BP below 140/90 mmHg for hypertensive patients.

Serum creatinine, glucose, total and high-density lipoprotein (HDL) cholesterol, triglycerides, were determined, while glycosylated haemoglobin (HbA1c) was only done on the diabetes patients. For diabetes, the ‘acceptable’ glycaemic target was HbA1c <7%.

The low-density lipoprotein (LDL) cholesterol levels were calculated. All participants had the required triglyceride level less than 4.5 mmol/L. The normal lipid profile used the following cut-off points: total serum cholesterol less than or equal to 5 mmol/L; LDL cholesterol level less than or equal to 3.0 mmol/L; HDL cholesterol more than or equal to 1.2 mmol/L; the ratio of HDLC/TC should be higher then _20% and not B20% triglyceride level more than or equal to 2.3 mmol/L.

Ten in-depth interviews were conducted with purposefully sampled nurses and doctors at six of the intervention clinics to examine their response to the intervention. These were conducted in private and lasted, on average, 45 minutes. The structure of the interview schedule was open and flexible, allowing respondents to explore issues in their own terms and to raise other topics for discussion.

The in-depth interviews were tape-recorded and transcribed verbatim. Each interview was individually analysed and coded. ‘Units of meaning’ in each sentence or paragraph were given descriptive codes or labels. The codes were then grouped into more abstract categories with various dimensions, and overall themes or core categories were identified. This analytical process followed the processes described by Strauss and Corbin (9).

### Statistics

Data were coded and computerised, and analyses were done in the SAS. Baseline tables of the randomisation groups were compiled which contained descriptive statistics, such as percentages, means, medians, and standard deviations. The impact of the intervention was assessed using an intention to treat analysis. For the primary outcome in the diabetic (HbA1c) and hypertension (systolic BP) cohorts, a linear regression model with an adjustment for the stratified design was used. Apart from the group and stratification main effects, the baseline value of the primary outcome for each participant was also included to improve precision. Standard errors and confidence intervals obtained from the linear regression models took account of the cluster design using robust cluster variance estimation. For secondary outcomes that were categorical, such as the process measures and the hypertension indicator, a binomial regression model was fitted to estimate risk differences and their 95% confidence intervals. The group and stratification variables were the main effects and the baseline measurement of the participant was used as a covariate. Standard errors were estimated using robust cluster variance estimation.

The cluster sizes of the two cohorts across the nine clinics of each arm were similar. Given the number of clusters in total (*n*=18) and adjusting for baseline differences, an individual-level analysis was carried out.

The Ethical Committee of the South African Medical Research Council provided ethical clearance for this project. The project protocol was registered retrospectively with the Pan African Clinical Trial Registry (www.pactr.org) with the following number: PACTR201303000493351.

## Results

### Baseline characteristics

There were 456 patients with diabetes (229 intervention and 227 control patients) and 920 patients with hypertension (461 intervention and 459 control patients) studied at baseline (Fig. 1 and Table 1). The mean ages, employment status, and gender distribution were similar among both the diabetes and hypertension control and intervention groups. The marked preponderance of women reflects the daytime service provided by these clinics. The control groups had more schooling than the intervention groups for both the patients with diabetes and hypertension. The majority of diabetes patients from both groups had type 2 diabetes and about 60% of these diabetic patients also had hypertension. There were 284 patients who had both diabetes and hypertension. Their data are included in both the diabetes and hypertension groups. For patients with both diabetes and hypertension, there were similar high prevalence rates of obesity, smoking, and elevated LDL cholesterol concentrations in the intervention and control groups.

**Table 1:** Baseline characteristics of the study cohorts (mean SD)

	Diabetes		Hypertension	
		
	Intervention group	Control group	Intervention group	Control group
No	229	227	461	459
Age (years)	58.1 (10.9)	58.6 (11.0)	59.5(10.9)	61.2(11.2)
Sex (M:F)	24.5:75.6	27.8:72.3	17.4:82.7	25.1:75.0
Employed (%)	18.8	23.8	15.6	21.4
Homemaker	22.3	16.7	20.6	15.3
Pensioner/disability grant (%)	45.4	49.3	45.1	50.5
Unemployed (%)	13.5	10.1	10.0	7.0
Educational status (%)				
No schooling	7.4	4.4	8.0	5.2
1–7 year (primary)	53.3	33.0	56.8	35.1
8–12 year (secondary)	39.3	62.6	35.1	59.7
Diabetes type				
Type 1	5.8	5.8	–	–
Type 2	91.6	91.1	–	–
Uncertain	2.6	3.1	–	–
Associated hypertension (%)	63.8	63.0	–	–
Associated diabetes	–	–	31.5	31.2
BMI (kg/m^2^)	31.0 (6.2)	30.3 (6.2)	31.7 (6.8)	31.2 (6.6)
Obese/overweight (%) BMI >25	83.7	80.3	84.0	82.4
Smoking (%)	54.5	48.9	54.9	50.5
Total cholesterol (mmol/L) (SD)	5.7 (1.4)	5.7 (1.4)	5.7 (1.2)	5.6 (1.3)
LDL cholesterol (mmol/L) (SD)	3.6 (1.1)	3.6 (1.0)	3.7 (1.0)	3.5 (0.98)
LDL cholesterol, >2.5 mmol/L (%)	84.8	82.8	89.0	84.9
HDL cholesterol (mmol/L) (SD)	1.0 (0.4)	1.1 (0.4)	1.1 (0.4)	1.1 (0.4)
Triglycerides (mmol/L) (SD)	2.5 (2.1)	2.7 (3.2)	2.0 (1.5)	2.2 (2.4)
Creatinine (mmol/L) (SD)	84.2 (37.7)	87.8 (63.2)	88.0 (41.1)	92.4 (60.4)

BMI=body mass index; LDL=low-density lipoprotein; HDL=high-density lipoprotein.

### Follow-up

The follow-up rates were calculated after incomplete data were excluded: for patients with diabetes intervention clinics (90.4%), control clinics (88.12%); and for the patients with hypertension intervention groups (93.1%) and control 88.9%. Twenty-six of the patients could not be traced 1 year after the baseline survey. Thirteen patients with diabetes and 16 with hypertension in the intervention clinics and 17 patients with diabetes and 38 with hypertension in the control clinics were excluded from the final analyses as they had incomplete data collection at follow-up.

#### Glycaemic control

The mean glycated haemoglobin level was similar in both groups at baseline and follow-up indicating a lack of effect of the intervention. Furthermore, the degree of glycaemic control was poor; at baseline 62.6% of patients at intervention clinics and 63.1% at control clinics had an HbA1c ≥7%. This did not change significantly at follow-up (Table 2).

**Table 2:** The use of the structured record at follow-up in the intervention clinics

	Diabetes (%) (95% CI)	Hypertension (%) (95%CI)
Number of patients’ folders evaluated at follow-up visits	*N*=214	*N*=429
Folders with structured records	58.1(32.7:82.9)	47.3(32.7:83.5)
Record present partially completed	56.8 (30.6:82.9)	46.9 (21.9:71.9)
Name and clinic number completed	35.6 (14:57.3)	44.5 (21.8:67.3)
Medical history recorded	31.9 (8.9:54.9)	25.5 (3.8:47.2)
Body weight recorded	20.1 (.01:42.5)	20.5 (.01:45.6)
Lifestyle education recorded	16.2 (0.01:34.5)	11.8 (0.01:27.1)
Fasting glucose recorded only once	3.9 (0.2:7.7)	–
Fasting glucose recorded twice or more	34.1 (9.9:58.2)	–
BP recorded only once	–	6.5 (0.9:12.1)
BP recorded two or more times	–	15.8 (3.5:27.6)
Foot examination recorded	23.1 (1:45.3)	–
Fundoscopy recorded	19.2 (6.3:32.1)	–
Proteinuria recorded	25.7(7.5:33.5)	22.7 (5.5:32.5)
Ketonuria recorded	8.7 (0.01:20.7)	–
HbA1c recorded	7 (0.01:19.9)	–
Total blood cholesterol recorded	10 (0.01:25.1)	6.3 (0.01:25.1)

#### Blood pressure control

At baseline, the BP was controlled (BP<140/90 mmHg) in 58.1 and 59.7% at the intervention and control clinics, respectively in the patients with hypertension. Once again, no significant improvement in BP control was recorded at either the intervention or control clinics at follow-up, again indicating a lack of the effect of the intervention (Table 2).

#### Process measures of clinical care

In the patients’ formal clinic records, there were no differences observed between intervention and control groups in the recording of process measures, such as foot examinations, visual acuity, or ophthalmoscopy examinations.

The use of the SR for patients with diabetes and hypertension is shown in Table 3. At follow-up, the SR record was found in the folders of only 58% of patients with diabetes and 47% of patients with hypertension at the intervention clinics (Table 2). Detailed data were infrequently recorded in the SR. For example, for patients with diabetes the fasting blood glucose was recorded more than once in only 38% of the SR and the HBA1c in only 7% of SR. For patients with hypertension, the BP was recorded once or more times in only 22.3% of SR.

**Table 3:** Results of the intervention trial

	Intervention		Control		
			
	Baseline	Follow-up	Baseline	Follow-up	Mean intervention effect (95% CI)
Diabetes					
Numbers	229	214	227	207	–
Median number of visits in the previous year	3	3	4	5	
Glycaemic control					
Mean HbA1c (%)	8.8	8.8	8.9	8.8	−1.0 (−1.1,–0.9)
% with HbA1c ≥7%	62.6	64.1	63.1	62.6	0.90 (0.53,–1.53)
Hypertension					
Numbers	461	429	459	408	
Median number of visits in previous year	2	4	3	4	
BP control					
Systolic BP mmHg (mean SD)	149.7 (26.3)	161 (28.9)	152.8 (27.1)	158.2 (29.5)	4.8(−1.3,–10.9)
Diastolic BP mmHg (mean and SD	87.5 (11.9)	88.1 (13)	86.6 (12.9)	87.1 (12.6)	0.93 (−2.07,–3.93)
Uncontrolled BP: HPT <140/90 mmHg or diabetes <130/85 mmHg	69.0	76.9	73.0	74.0	1.3 (0.83,–2.04)
Process measures recorded					
Opthalmoscopy in HPT (%)	16.0	19.0	18.0	18.0	3.0 (−14,–11.7)
Ophthalmoscopy in diabetics (%)	18.0	14.4	9.0	3.5	1.5 (−15,–13.5)
Visual acuity in diabetics (%)	6.0	18.4	5.0	12.0	7.4 (−13,–27)
Foot examination in diabetics (%)	13.1	28.9	9.3	15.1	10.1 (−16.3,–36.4)

#### Analysis of in-depth interviews with doctors and nurses at the intervention clinics

The in-depth interviews revealed that both doctors and nurses were carrying out enormous workloads at the CHCs. This was due to an acute shortage of staff while patient numbers were also increasing as a result of policy changes, which directed growing numbers of patients to seek healthcare at the CHCs. This was compounded by the flow of patients from rural areas. Budget constraints impacted negatively on the availability of necessary investigations and other healthcare resources. This ever changing and demanding work environment resulted in many staff experiencing stress, frustration, and low levels of motivation. Table 4 provides the responses of the healthcare providers at the CHC to the intervention study.

**Table 4:** Doctors’ (Drs) and Nurses’ (Nrs) responses to the intervention of the structured records with prompts (SR) determined during in-depth interviews

Topic	Themes	Quotation (Interview number)
Attitude to research and researchers	Drs and Nrs well disposed to researchers. Saw no benefit for themselves, preferred own notes. SR perceived as research tool.	‘The staff responded positively to it (SR). They were quite enthusiastic’ (1). ‘Most of the forms were completed and there was little resistance to complete the forms’ (4). ‘So it (SR) didn't really change our management or treatment of patients’ (5). ‘We used it so that they could do the research’ (7). ‘It was just for the study's purpose really, it wasn't really for my purpose in the end’ (9).
Extent of implementation of intervention	SR only used for a few months	‘Many patients did not have it in their folders, because of the workload’ (2). ‘I think most of the patient's folders have got the forms in, but I don't think that it has been carried through the way they (researchers) wanted it to be’ (12).
Problems experienced with the SR	Time-consuming duplicate record keeping with high patient loads. SR had no space for additional note keeping. Patient counselling is too time consuming. SR will not be seen as a legal document. Special investigations required are too costly.	‘So the workload is horrendous. That's a serious factor’ (1). ‘He just found it too much of a rate limiting exercise. It was slowing him down too much’ (2). ‘I was very pleased when it came to an end. I felt committed because I had said I would do it’ (8). ‘It was just too laborious, you know just duplicating things and not taking into account all the other things that go wrong with the patient’ (9). ‘Many of outpatients are not that simple, they are not just hypertensive. The great majority have got a combination of problems and there is no space on these forms to make note of that’ (9). ‘You know if you have to go to court, for something, they want to see your notes. This (SR) is not a legal document’ (8).
Perceived benefits of the research	Drs and Nrs communicated better. Useful to have all relevant information in one document. Patients learned more about their conditions. Drs found records confirming the protocols they already used. Prompted more regular follow-up of patients. Prompted to look for complications more frequently.	‘It (SR) did teach me some things to look for. I mean the other thing which I never used to do was the ventricular enlargement. Now I try to do that’ (7). ‘You can just quickly look there and see everything visually’ (8). ‘On this (SR) I could see when I had done it, otherwise you find that people do get left out. You know, you find suddenly that, ooh – I haven't done this for two years. So it prompted me’ (7). ‘The form (SR) is a quick form to screen the patient- how well is he controlled, how is the medication, what target organ disease does he have and what screening methods did you have the previous year or two, especially in a system like the day hospital where sometimes other doctors have to see your patients’ (6).

Given the demands on them, the staff members were remarkably positively disposed to the researchers and willing to incorporate the intervention into their daily activities. This occurred despite the doctors’ not perceiving the SR to be particularly useful and their preference for their own clinical notes on the patients. The staff saw the SR purely as a research tool and felt that it did not change their management or treatment of their patients. In fact, they felt that the information in the SR confirmed the treatment protocols that they had been following in the past.

Despite the goodwill they had towards the researchers, their excessive workload undermined their ability to complete the data required on the SR. The fact that they ended up spending extra time duplicating patient's data in the SR, as well as in their own notes in the patient's folders, was the main reason why they could not sustain the intervention. Nurses and doctors reported that many folders had no SR in them and often, if present, they were only partially completed.

Some of the perceived benefits of using the SR for the staff included the usefulness of having all the relevant information in one easily accessible document. The nurses in particular felt that the patients learned more about their chronic conditions as they were receiving more health education from the staff. Some doctors admitted that the SR did prompt them to screen for complications more frequently and to follow patients up more regularly.

## Discussion

This RCT of a multifaceted intervention incorporating educational outreach and an SR in which the national guidelines for the managing of people with type 2 diabetes and hypertension were embedded, failed to demonstrate any benefit in the primary care clinics where it was tested. The most plausible explanation for this finding is the evidence that the SR was found in less than 60% of all patients’ folders. Though when present, the SR was seldom used, suggesting that the trial did not actually evaluate the usefulness of this record. The lack of implementation of the intervention could also explain these findings.

A number of factors are likely to have contributed to the poor implementation of the intervention. The trial was conducted at a time of major restructuring of the health services in South Africa. The new South African government came to power in 1994 and embarked on a systematic process of health sector reform, moving away from a hospital-centred curative-based health system to one based on the Primary Health Care (PHC) approach. The District Health System (DHS) was subsequently formally established through the National Health Act of 2003 (Health Act) (10). The DHS from its inception was involved in a well-recognised health sector reform process, usually coupled with a decentralisation process of moving healthcare management from the central to peripheral levels of government. The proposed plan intended that 90% of patient contacts within the health system should take place at the primary care level and CHCs, with the remainder requiring more specialised intervention at regional or provincial hospitals.

The redistribution of patients to the primary level resulted in increasing patient numbers, but this was unaccompanied by increases in staff numbers and clinic facilities. Indeed, there were acute staff shortages and many vacant posts were left unfilled at the time when this study was conducted. Therefore, it was not surprising that the staff described working conditions in the public sector as being extremely demanding and difficult. The Government's apparent lack of concern to effectively resolve these problems left staff at the CHCs feeling angry, frustrated, and despondent about their work situation (11).

Although the SR with the embedded national guidelines made recommendations that constituted good clinical practice, they did not take budgetary restraints or the contexts in which the staff were working into consideration. For example, the SR included an annual glycated haemoglobin testing, which, if requested for all the diabetes patients, would have consumed the centre's entire allocation for biochemical investigations. An ECG was recommended as part of the annual evaluation, but the equipment was not available at all CHCs or in many instances, the staff did not have the time to perform this investigation. As the staff felt these recommendations were idealistic and not feasible in their demanding work situation, this induced a sense of frustration, alienation, and in certain cases rejection of the guidelines. However, some doctors reported that they felt that their practice already included many of the recommendations of the guidelines, and as such what was offered was not new.

The SR was thought to contain some valuable elements, such as the algorithms for pharmacotherapy for glycaemic and BP control, the provision of a ‘flow sheet’ for the recording of blood glucose and BP measurements at the routine visits and the reminders to look more closely for complications of their patients’ conditions. However, doctors preferred to have much more space to write their notes and were concerned that the SR mitigated against holistic patient care due to the sole focus being on hypertension and diabetes. The insights gained from the in-depth interviews highlight the importance of a much greater degree of involvement of the end users in the design of such interventions. It is quite conceivable that the staff would have found a much simpler intervention more acceptable. This could take the form of a simple stamp with the most important components of care, such as BP, urine testing, blood glucose, and processes for screening for complications placed in their routine records.

The interviews revealed a remarkable degree of goodwill of the healthcare providers towards the researchers. However, if the work environment is as demanding as found in these settings, then doctors and nurses cannot maintain efforts to evaluate new interventions, particularly if they are not found to be useful and demand extra time from busy staff.

Although this study was conducted in 1999 and 2000, the value of publishing data on a study with negative outcome this long after data collection, may well be questioned. However, we feel that the study illustrates important requirements for policy implementation in developing countries in overextended resource-scarce settings, as described above, which may be of value. Limited data on such studies, particularly in developing country settings are available. Furthermore, the current international drive to publish RCTs, even negative ones, to ensure comprehensive availability of evidence-based data provides a cogent motivation for submitting this article.

This unsuccessful evaluation of the intervention emphasises the need to formulate clinical guidelines for chronic diseases that can realistically be implemented in resource-scarce primary healthcare settings present in most developing countries. The introduction of these guidelines needs to be accompanied by organisational changes to ensure that drug and investigational recommendations are feasible.
